# Reimmunization increases contraceptive effectiveness of gonadotropin-releasing hormone vaccine (GonaCon-Equine) in free-ranging horses (*Equus caballus*): Limitations and side effects

**DOI:** 10.1371/journal.pone.0201570

**Published:** 2018-07-31

**Authors:** Dan L. Baker, Jenny G. Powers, Jason I. Ransom, Blake E. McCann, Michael W. Oehler, Jason E. Bruemmer, Nathan L. Galloway, Douglas C. Eckery, Terry M. Nett

**Affiliations:** 1 Animal Reproduction and Biotechnology Laboratory, Department of Biological Sciences, Colorado State University, Fort Collins, Colorado, United States of America; 2 Biological Resources Division, National Park Service, Fort Collins, Colorado, United States of America; 3 Department of Ecosystem Science and Sustainability, Colorado State University, Fort Collins, Colorado, United States of America; 4 Theodore Roosevelt National Park, National Park Service, Medora, North Dakota, United States of America; 5 National Wildlife Research Center, Wildlife Services, Animal and Plant Health Inspection Service, United States Department of Agriculture, Fort Collins, Colorado, United States of America; University of Tasmania, AUSTRALIA

## Abstract

Wildlife and humans are increasingly competing for resources worldwide, and a diverse, innovative, and effective set of management tools is needed. Controlling abundance of wildlife species that are simultaneously protected, abundant, competitive for resources, and in conflict with some stakeholders but beloved by others, is a daunting challenge. Free-ranging horses (*Equus caballus*) present such a conundrum and managers struggle for effective tools for regulating their abundance. Controlling reproduction of female horses presents a potential alternative. During 2009–2017, we determined the long-term effectiveness of GnRH vaccine (GonaCon-Equine) both as a single immunization and subsequent reimmunization on reproduction and side effects in free-ranging horses. At a scheduled management roundup in 2009, we randomly assigned 57 adult mares to either a GonaCon-Equine treatment group (*n* = 29) or a saline control group (*n* = 28). In a second roundup in 2013, we administered a booster vaccination to these same mares. We used annual ground observations to estimate foaling proportions, social behaviors, body condition, and injection site reactions. We found this vaccine to be safe for pregnant females and neonates, with no overt deleterious behavioral side effects during the breeding season. The proportion of treated mares that foaled following a single vaccination was lower than that for control mares for the second (*P =* 0.03) and third (*P* = 0.08) post-treatment foaling seasons but was similar (*P* = 0.67) to untreated mares for the fourth season, demonstrating reversibility of the primary vaccine treatment. After two vaccinations, however, the proportion of females giving birth was lower (*P* <0.001) than that for control mares for three consecutive years and ranged from 0.0–0.16. The only detectable adverse side effect of vaccination was intramuscular swelling at the vaccination site. Regardless of vaccine treatment (primary/secondary), approximately 62% (34/55) of immunized mares revealed a visible reaction at the vaccine injection site. However, none of these mares displayed any evidence of lameness, altered gait or abnormal range of movement throughout the 8 years they were observed in this study. Our research suggests that practical application of this vaccine in feral horses will require an initial inoculation that may provide only modest suppression of fertility followed by reimmunization that together could result in greater reduction in population growth rates over time.

## Introduction

Anthropogenic disturbance of landscapes and natural resources is pervasive across much of the earth, resulting in increased conflict between humans and wildlife and a need for effective resource management [1]. Humans indeed have tried to control animal abundance in some capacity for over 13,000 years [2]. Regulating abundance of wild animals using fertility control or contraception is a relatively new development, emerging only 50 years ago [3]. Such tools are appealing to wildlife managers and stakeholders because they present a non-lethal solution for regulating abundance when species pose a risk to human interests and safety, and when wildlife densities are high enough to disrupt ecosystem function [4,5].

Feral horses (*Equus caballus*) present perhaps one of the most unique wildlife management problems worldwide. Humans have spent centuries propagating and dispersing domestic horses to every continent except Antarctica over the last several centuries, only to have inadvertently created expansive feral populations that now compete with humans, wildlife, and domestic animals for resources [6]. The unique relationship between humans and horses has resulted in a precarious dichotomy, with the struggle for relief from conflict and resource competition challenged by a mutualistic societal view where feral horses are perceived as part of our social environment. This struggle is elevated in the United States, where federal law (*P*. L. 92–195, as amended) provides protection for feral horses and burros (*Equus asinus*) on large expanses of public land, and establishes guidance for their management as a wildland species [7].

Current methods of population control for free-ranging horses in the U.S. involve periodic removals and adoption or sale of surplus animals, or maintaining excess animals in long-term holding facilities which are expensive, resource intensive, and unsustainable [8]. Clearly, more efficient, cost effective, and humane approaches to reducing feral horse densities on public lands are needed. Controlling the fertility of female horses offers a potential complementary or alternative strategy for limiting the growth of some populations [9].

A promising immunological approach to contraception in feral horses and other wild ungulate species involves immunization against gonadotropin- releasing hormone (GnRH), a small neuropeptide that performs an obligatory role in mammalian reproduction [10]. When conjugated to a highly immunogenic carrier protein and combined with a potent adjuvant, GnRH vaccination actively stimulates a persistent immune response resulting in prolonged antibody production against endogenous GnRH. These antibodies induce transient infertility by binding to GnRH, thus preventing attachment to receptors on pituitary gonadotropes, suppression of gonadotropin release, and ultimately ovulation in females [11, 12]. As anti-GnRH antibodies decline over time, the availability of endogenous GnRH increases and treated animals generally regain normal fertility [13–17].

The GnRH-based contraceptive agent known as GonaCon-Equine (National Wildlife Research Center, Fort Collins, CO, USA; [18] is registered by the United States Environmental Protection Agency as a restricted-use pesticide for contraception of adult female feral horses and burros. A single immunization with this or earlier versions of this vaccine (more generally referred to as GonaCon) have been shown to induce extended infertility (≥ 2 yr) in numerous wild ungulate species including captive and free-ranging elk (*Cervus elaphus*) [15–17] white-tailed deer (*Odocoileus virginianus*) [18–20], bison (*Bison bison* [21]), and feral horses [22– 24]. However, multiple years of infertility are only experienced in a fraction of vaccinated animals. In free-ranging elk, for example, there was approximately a 90% treatment effect the first year after vaccination but this declined to 50% by the second year; with no measurable effect by year three [16]. Similar declines in effectiveness have been reported for captive feral horses treated with the same vaccine [22].

Booster vaccinations generally result in a more profound and longer-lasting antibody production due to the anamnestic (cell memory) response [25]. Traditional veterinary vaccinology suggests that non-replicating vaccines most often require two initial doses 2–6 weeks apart followed by booster vaccinations every 1–3 years [26]. Repeat immunizations using a variety of GnRH vaccines in domestic horses improves contraceptive efficacy and suppress behavioral and physiological estrus [27–29]) However, these GnRH vaccines differ from GonaCon-Equine in that they incorporate different protein carrier molecules and adjuvants, and are formulated for short duration (< 1 yr.) effectiveness. They are also administered on a more traditional vaccination schedule with a primary set of immunizations followed by periodic boosters.

Other forms of wildlife fertility control vaccines have adopted comparable initial and booster recommendations [30–32]. However, this intensive vaccination schedule places significant logistical barriers on application in free-ranging animals. GonaCon vaccine is formulated with highly immunostimulating mycobacteria as a component of the adjuvant. This may prolong the initial and subsequent booster vaccination windows for optimum efficacy as initial antibody concentrations are maximal 2–12 months post-primary vaccination [15]. GonaCon vaccine is one of the rare exceptions among animal vaccines in that the formulation initiates high antibody titers that remain elevated in some individuals after a single-injection; however, little research has been conducted to evaluate booster doses of this vaccine in any free-ranging wild ungulate [17, 24] or domestic species [33]. While a single immunization against GnRH may be preferred from a practical perspective, there may be a more optimal vaccination schedule that balances the need for minimizing animal handling or contact while maximizing vaccine effectiveness. Thus, it’s imperative to investigate the safety and long-term effectiveness of repeat vaccination and to evaluate its potential to limit fertility in this long-lived and perennially pregnant species.

In female wild ungulates, adverse side effects following a single immunization against GnRH appear to be minimal. Evaluation of biological side effects has been reported for numerous wild ungulate species including white-tailed deer [13, 34], elk [15, 16, 35], feral pigs [36], bison [21], and free-ranging horses [17, 24]. A summary of results from these investigations indicate that GonaCon is reversible, safe for use in pregnant females, does not significantly change social behaviors [37] or negatively affect neonatal development, survival, or maturation [15, 35]. No adverse effects of vaccination have been shown to be related to general health, body condition, blood chemistry parameters, or hematology of treated animals. The most apparent pathological side effect has been the development and persistence of non-debilitating granulomatous and often purulent inflammation at the site of injection. In all studies, where post-mortem examinations have been conducted, injection-site lesions were pervasive, but in some species, such as white-tailed deer and elk, they were not apparent antemortem. Likewise, in cases where injection-site reactions have been documented, no clinical evidence of lameness, impaired mobility, or depression, have been reported [13, 15–17, 21, 24, 34, 35].

While documentation of contraceptive efficacy and side effects of GonaCon have been described for a variety of wild ungulates, similar evidence for feral horses is limited. To our knowledge, only two long-term (≥ 3 years) empirical investigations have been conducted using GonaCon-Equine. These include a clinical trial with captive feral mares [22] and the other with free-ranging mares in a natural environment [23]. In the study with free-ranging horses, vaccination significantly reduced foaling rates of treated females, however, effectiveness was inconsistent over time and was substantially lower than that reported for captive feral mares treated with the same vaccine [22]. Furthermore, neither of these studies integrated revaccination as a strategy to increase vaccine efficacy. Lastly, these inquiries provide little quantitative evidence of the reversibility of the effects of this vaccine, the presence or absence of adverse side effects related to inoculation of pregnant mares, and neither examined the potential for increased side effects with reimmunization.

Knowledge of the effects of GonaCon-Equine on equid fetal health, neonatal survival, and body condition is largely anecdotal, whereas injections site reactions to booster immunization and the efficacy of revaccination are limited to two investigations [24, 33]. Clearly, additional research is needed to further define the long-term therapeutic effectiveness and contraindications of this potential technology before resource managers can make informed decisions regarding its practical application for stabilizing the growth rate of free-ranging feral horse populations.

Consequently, the fundamental objectives of this investigation were: 1) to determine the duration, effectiveness, and reversibility of both a single immunization and subsequent reimmunization against GnRH in suppressing reproductive rates of free-ranging mares in a natural environment, 2) to determine the safety and adverse side effects (if any) in free-ranging mares including assessment of general health, body condition, effects on current pregnancy, injection site reactions, and neonatal health and survival and, 3) to compare the effects of a single vaccination against GnRH on time budgets and social behaviors [37] to similar behaviors following reimmunization. Based on evidence from prior studies with feral horses and other wildlife species, we predicted (H_1:_) that a single vaccination against GnRH would suppress fertility for multiple years with decreasing effectiveness over time but would not result in permanent infertility. Furthermore, we surmised (H_2:_) that the anamnestic immune response to revaccination would be more effective and longer lasting in suppressing fertility than the initial immunization alone. Moreover, we reasoned (H_3:_) that except for localized inflammatory reactions at the injection site, we would not observe other adverse side effects (i.e. lameness, detrimental effects on existing pregnancy, neonatal health and survival, body condition, behavioral changes). Apart from determination of return to normal fertility of treated mares, these objectives and hypotheses were addressed and accomplished in this investigation.

## Materials and methods

### Study area

We conducted this research in the South Unit of Theodore Roosevelt National Park (THRO), USA) (45° 55’N/103° 31’W). This unit is located near the town of Medora in southwestern North Dakota and encompasses approximately 19,000 ha of native vegetation. The landscape is topographically diverse and consists of eroded badlands with gullies and ravines separated by relatively large upland plateaus and small erosion-resistant buttes capped by scoria. Elevation ranges from 683 m to 870 m. Its continental climate is characterized by short, arid summers (mean temperature 21^0^ C) and long, cold winters (mean temperature -12^0^ C) [38]. Precipitation is irregular in amount and distribution with a long-term annual mean of 38 cm with most of this falling as rain showers from April to June [39].

Vegetation is primarily mixed-grass prairie dominated by needle-and -thread grass (*Hesperostipa comata)*, western wheatgrass (*Pascopyrum smithii)*, threadleaf sedge (*Carex filifolia)*, blue gramma (*Boutelous gracilis)*, and little blue-stem (*Schizachyrium scoparium)*. Cottonwood (*Populus deltoides)* gallery forests occur along perennial water courses while hardwood stands of green ash (*Fraxinus pennsylvanica)* and chokecherry (*Prunus virginiana)* dominate the upland drainages. Dense stands of Rocky Mountain juniper (*Juniper scopulorum*) are common on steep north-facing slopes [40].

Besides feral horses, sympatric wild ungulate species include bison, elk, mule deer (*Odocoileus hemionus)*, white-tailed deer, and pronghorn (*Antilocapra americana)*. Horses and bison are confined to the South Unit of the Park by a 1.8–2.4 m woven-wire boundary fence. Currently, horse numbers are controlled through periodic live capture and removal of select individuals. Free-ranging horses at THRO are classified by the National Park Service (NPS) as “feral livestock” and managed as a “historical demonstration herd”. The most recent estimate of population size (2017) is 150–175 horses and the Park has set a management goal for this herd at approximately 50–90 animals.

The social structure of this population consists of 14–16 social groups (bands) that include a single dominant stallion, subdominant stallions, and 1–5 adult mares, yearlings, and foals of both sexes. Males greater than 1 year of age that have not acquired a band are usually found in ephemeral bachelor groups of 3–6 individuals. These bands are non-territorial and are spatially distributed across the South Unit primarily east of the Little Missouri River. All horses are known by unique coloration and markings and have been previously identified and assigned individual identifiers by managers. Photographs of each animal from birth to adulthood assist in the identification of individuals. Age, reproductive history, and genealogy data for each animal has been maintained since 1993.

In spring/summer 2009, we collected pre-treatment data on all mares and bands within THRO. The purpose of this effort was: 1) to determine the sample size and sampling intensity required to achieve acceptable statistical power (≥ 80%) to detect fixed differences (≥ 50%) in foaling proportions of experimental groups, 2) to assess unknown logistical limitations of locating and identifying specific study mares within bands of horses, and 3) to train field technicians to observation protocols, and collect pre-treatment time budget and social behavioral data.

### Experimental animals and treatments

#### Primary vaccination (2009–2013)

During a scheduled management roundup at THRO (18–23 October 2009), 160 horses were guided by helicopter into permanent corrals and handling facilities. An attempt was made to capture the entire population to maximize sample sizes for this research project and to remove excess horses to meet desired herd management objectives. A total of 57 adult mares (2–17 years of age) and associated foals, and band stallions, were captured, identified, treated, and retained in the Park for this experiment. Using a randomized complete block design, we established two experimental groups consisting of a GonaCon-Equine treatment group (*n* = 29) and a saline control group (*n* = 28). Mares were paired (blocked) based on age and pregnancy status such that animals within a block were as similar as possible. Within each block, individual mares were then randomly assigned to either a control or treatment group.

Equine veterinarians and a reproductive specialist, blinded to treatment status, assessed the general health, body condition, pregnancy status, and approximate gestational stage of each mare. We determined pregnancy status and gestational age by transrectal palpation and ultrasonography of the reproductive tract [41]. We collected whole blood (up to 50 mL) via jugular venipuncture (BD Vacutainer SST; Becton Dickinson and Co., Franklin Lakes, NJ) then centrifuged these samples at the capture site, and temporarily stored serum in cryovials at -20^0^ C. We later transferred frozen serum on dry ice to Fort Collins, Colorado, where it was stored at -80^0^ C. We also assessed serum for exposure to common pathogens known to cause abortions in horses (e.g. equine herpesvirus-1, equine infectious anemia, equine viral arteritis and contagious equine metritis) that could confound the interpretation of treatment-induced infertility [42].

We applied treatments while mares were restrained in a squeeze chute. Females in the treatment group received an intramuscular injection in the lower left gluteal musculature, by hand-held syringe (18-gauge, 3.8 cm needle) containing GonaCon-Equine (2.0 mg GnRH conjugate + adjuvant; 2.0 mL). The vaccine contained multiple synthetic copies of GnRH coupled to a large immunogenic carrier protein (Blue Carrier; Biosonda, Santiago, Chile) that was combined with a water-in-oil adjuvant containing killed *Mycobacterium avium* ssp. *avium* (AdjuVac, National Wildlife Research Center) [18]. Mares in the control group were injected in a similar manner, with an equal volume of physiologic saline solution (0.9% NaCl; 2.0 mL). We chose to inject the vaccine into the gluteus muscle (~ 15 cm distal to the point of the hip) rather than the neck because of greater safety for hand-injection, enhanced detection of potential injection site reactions under field conditions, and the preferred location for potential remote dart delivery of the vaccine.

#### Secondary vaccination (2013–2017)

Four years later, during 23–25 September 2013, we similarly rounded up the entire THRO horse population and moved and handled them through existing corrals and chute systems to remove excess animals from the Park. Given this unique opportunity and endorsement from the Park, we retained all available mares previously immunized and control mares, retreated them, assessed pregnancy status, and determined body condition using techniques identical to those applied at the 2009 roundup. Two mares in the control group and 4 mares in the treatment group died between 2009–2013 and therefore, were not available for this experiment. We attributed these mortalities to malnutrition, dystocia, broken appendage, and unknown causes not related to treatments. The one exception in our 2013 protocol was that we injected the booster vaccination into the opposite (right) hip from where the primary (left hip) vaccination was previously administered. This provided the opportunity to simultaneously evaluate injection site reactions related to both immunizations. Treatment mares again received 2.0 mL GonaCon-Equine and control mares 2.0 mL saline.

### Field measurements

Using 2–3 trained technicians and occasional equally trained volunteers, we conducted field measurements and observations consistently from year to year. Prior to field observations, technicians were provided with photographic images of individual horses and required to recognize them by band association, natural markings, and pelage coloration. They were also trained or had previous experience in identifying prepartum characteristics of pregnancy (e.g., enlarged abdomen, mammary gland development, waxing teats, behavior, etc.), as well as, body condition scoring, and the appearance and classification of injection site reactions to the vaccine. We collected all data from ground surveys (foot, vehicle, horseback) using binoculars and spotting scopes. Although technicians were unaware of treatment assignments of individual mares, the presence of injection site reactions in several GonaCon-treated mares could have revealed their treatment designation.

#### Reproduction

We predicted that pregnant females inoculated with GnRH at the fall gathers of 2009 and 2013 would give birth to a healthy foal the following spring (2010 or 2014) and presumably be infertile during subsequent breeding seasons. Thus, the effects of the primary or booster vaccinations on reproduction (foaling proportions) would not be observed until the 2011 and 2015 foaling seasons, respectively. These are the first breeding seasons that a treatment or retreatment effect on mare fertility could be detected when using foaling observations to assess successful contraception by the vaccine.

We determined the effectiveness, duration of effects, and reversibility of the primary and booster vaccinations on reproduction by comparing foaling proportions of treated and control mares during 1 March to 31 December 2009–2017. We chose to use the term vaccine “effectiveness” rather than “efficacy” because it more realistically represents how GonaCon-Equine affects fertility under more natural field conditions compared to a controlled clinical trial [43, 44]. We defined vaccine effectiveness (VE) as the proportional reduction in annual foaling (F = number of mares with a foal/ total number of mares in a treatment group) between control and treated mares. Vaccine effectiveness is equivalent to relative risk reduction (RRR) in medical statistics and was calculated from the risk ratio $\left(RR=\frac{F_{Trt}}{F_{Con}}\right)$ where F_*Con*_ = foaling proportion of the control mares, and F_*Trt*_ = the foaling proportion of the treated mares. Risk ratio was calculated using the *fmsb* package in program R [45–47] and we then solved for VE as follows:

$$VE=\frac{F_{Con}-F_{Trt}}{F_{Con}}=1-\frac{F_{Trt}}{F_{Con}}=1-RR$$

Each year of the study, we estimated annual foaling proportions by locating all bands to identify individual mares and determine the presence or absence of foals. During the intensive sampling period (1 March–1 August), we attempted to observe 95% or greater of all experimental mares and foals (when present) at least weekly and 100% of them every two weeks, then opportunistically until 31 December. We did not attempt to assess contraceptive effect based on visual characteristics of pregnancy but did use these criteria to prioritize weekly observations of individual mares. Instead, we defined foaling as a parturition event or neonatal foal by side, as detected by direct observation. We matched foals with dams through observations of nursing and repeated close association during feeding, bedding, and traveling [48, 49]. We collected neonatal data at first sighting of a foal and estimated date of birth by observing the foal’s level of activity, presence of an umbilicus, and elapsed time since the dam was last observed pregnant [50]. We photographed and estimated the age of each new foal when first observed, recorded its sex, general health (vigorous, average, poor), markings, and band association, and gave it a unique identifier; then entered these observations into a herd database. Finally, we assessed the utility of using foaling proportions as a proxy for pregnancy proportions by comparing pregnancy proportions determined at the time of each gather in 2009 and 2013 to foaling proportions observed in 2010 and 2014.

### Side effects

#### Behavioral

We repeated thee behavioral measurements with the same treatment groups of mares that were previously conducted during an earlier phase of this project [37]. We proposed that, if greater contraceptive effectiveness after reimmunization against GnRH was achieved, it would potentially provide a larger and more statistically powerful sample size of contracepted animals in which to detect behavioral changes related to this vaccine (if they occurred).

We completed an intensive behavioral study during an earlier phase of this larger GnRH study during 2009–2010 [37]. To make these analyses directly comparable to that prior study, we followed the same behavioral sampling design as that described previously [50]. Briefly, this included blocking observations into three daylight time periods (08:00–12:00 h, 12:01–16:00 h, and 16:01–20:00 h) with observations conducted during the primary breeding season, 1 March– 1 August 2014. Each observation session included collection of a 20-min instantaneous scan sample of time budgets at 1 min intervals for each adult band member (≥1 year old), and all-occurrence data collection for social interactions [37].

Primary behavior categories included feeding, resting, locomotion, maintenance, and social behaviors [51]. Social behavior data included herding, reproduction, agonism, harem-tending, and harem-social behavior, and were collected at all occurrences throughout the observation sessions. Harem-social behavior was not collected through all-occurrence sampling in our previous study; however, it was collected during the scan sample in the previous study and was worthy of further consideration here. We defined this category as interactions between two individuals that did not meet the definition of the other all-occurrence behaviors (e.g. allogrooming and non-reproductive olfactory investigation).

We observed all horses from the nearest distance that did not elicit attention to the presence of the observer, typically 50–200 m. All observations were conducted using a 15–45 × 600 mm spotting scope or 10 × 42 mm binoculars when the distance between horses and observers was too far to allow unassisted detailed observation. We observed each band of horses weekly or bi-weekly in conjunction with other field assessments.

#### Physiological

Concurrently with foaling and behavior observations, we evaluated and compared potential adverse side-effects of treatment on injection-site reactions, body condition, success of existing pregnancy, and neonatal survival in treated and control mares. We made assessments of these potential side-effects monthly during the primary foaling season and opportunistically for the remainder of the year. We observed each mare for the presence or absence of visible lesions, swellings, or discharge at the injection site. In addition, we documented evidence of lameness (e.g. limping, gait alteration, reluctance to stand or bear weight on a limb), as well as behavioral depression, muscle tremors, or other systemic reactions that could be related to the vaccine treatment. We classified injection-site reactions according to the following criteria: 1) **abscess**–an open sore usually with fluid drainage or discharge, 2) **swelling**–a raised area of tissue of variable size and shape with no visible fluid drainage, 3) **lameness–**any abnormal range of movement or stiffness in the leg where the vaccine injection was delivered, 4) **none–**no observable reaction [52]. These categories were not mutually exclusive with respect to a single observation and both sides of the animal were observed, when possible. For these observations, we approached as near as possible to individual horses (≤ 50 m) and assessed and photographed each injection-site reaction for later evaluation. At the same time, we visually evaluated body condition of each mare and scored condition as previously described [53]. We evaluated the success of the existing pregnancy by comparing foaling proportions between treated and control groups in 2010 and 2014. We measured neonatal survival as the proportion of foals surviving to 14 days of age and post-natal survival to 200 days.

### Statistical analysis

#### Reproduction

Yearly foaling data are reported as the proportion of mares observed with a foal in each group. We used asymptotic approximation to the binomial distribution to compute 95% confidence intervals for these proportions using package *binom* in program R [45, 47]. We used a risk ratio analysis (α = 0.05) to compare all observed annual proportions between treatment groups. We used the same method to evaluate the success of the existing pregnancy between groups during the first foaling season post-vaccination (2010 and 2014). All comparisons between treatment groups were made within a single year and without multiple testing corrections.

#### Behavior

We used the same statistical approach for the analyses in 2014 as that used in 2010 [37]. We modeled the frequency of each behavior using mixed-effects linear regression, where individual female identity and sampling time (time of day) were included as random effects on the intercept term of each model. This accounted for variation that may have been present among individuals who were sampled repeatedly, though not always equally over time, and for temporal variation in behavior when samples were not equally collected across all times of the day. Time budget behaviors sampled at 1-min intervals were aggregated into proportion of time spent per behavior to calculate an independent measure of behavior per observation session. We used the *lme4* package of R version 3.1.2 (The R Foundation for Statistical Computing 2014) and SYSTAT 12.02.00 (SYSTAT Software, Inc. 2007) to calculate descriptive statistics and obtain mixed-effects model estimates using restricted maximum likelihood [54]. Separate models were fitted for each time budget behavior with the fixed effects of treatment group (treated or control), foal presence (dependent foal < 1 year of age present with the female, or no foal present with the female), female age, and band size. In the previous study, we considered band fidelity (number of times a female moved bands within a year), but data were too homogeneous to consider that factor in 2014: only 8 horses moved bands at all (4 treated/4 saline) and five of those moved collectively to a different stallion.

#### Physiological

We used descriptive statistics (arithmetic means with ± 95% CI) to compare, occurrence of lesions at the injection site and 1-tailed Fisher’s exact test (α = 0.05, 1df) to compare foal survival proportions of treated females to that of controls. We used normal binomial distributions to compute confidence limits for the differences between proportions using Jeffrey’s interval for small sample sizes [55]. Effects on body condition scores were examined using generalized linear models in the *lmer* package in program R [56]. We employed random effects for year and individuals and then compared this nested model to full models which added the effect of either treatment or foaling using an ANOVA.

This research was approved by the Institutional Animal Care and Use Committees of the National Park Service (NPS) (Permit Numbers: MWR_THRO_Baker_Horse_2013.A3, MWR_THRO_Baker_Horse_2015.A3) and Colorado State University (IACUC Protocol No. 17-7651A). This study was conducted in accordance with good laboratory practices (GLP) and oversight from United States Department of Agriculture/National Wildlife Research Center (No.QA1647). All data collections were conducted after obtaining a scientific collection permit issued by Theodore Roosevelt National Park (THRO-2010-SCI-0010). All work, other than animal handling and vaccination at the two feral horse roundups, was observational. Every effort was made to prevent and minimize disruption of natural band dynamics and individual horse behavior and well-being during handling and treatment application.

## Results

The statistical process used to select experimental mares for this investigation resulted in two treatment groups that were relatively homogeneous in age, body condition, body mass, and pregnancy status [S1 Table]. Results of pregnancy assessment indicated that most mares were pregnant at the 2009 (0.86 (49/57), 95% CI = 0.74–0.93) and 2013 (0.90 (46/51), 95% CI = 0.79–0.96) roundups, thus providing sufficient opportunity to evaluate and compare the safety and potential side effects of vaccine treatment on pregnancy and neonatal survival.

Transrectal ultrasonography revealed that the fetuses of most pregnant females were approximately 120+ days old at the roundup and that most had descended over the pelvic rim preventing a more accurate assessment of gestational age at treatment application [57]. To provide a more precise estimate, we used an estimated gestation period for horses of 342 days [58] and the approximate foaling date (± 5 days) of each mare in 2010 and 2014 and then back-calculated to the date of treatment application at the 2009 (18–23 October) and 2013 (23–25 September) roundups. Using these calculations, we estimated mean gestational age at vaccine inoculation in 2009 to be 162 days (95% CI = 150–175) for treated mares and 154 days (95% CI = 138–170 days) for control mares. For 2013, we used the same calculation and projected that, on average, females were reimmunized against GnRH at approximately 129 days (95% CI = 105–151 days) of gestation and saline-treated control mares at 132 days (95% CI = 119–144 days).

Following the 2009 and 2013 roundups and release, experimental mares distributed themselves among 16–19 individual bands. At least one treated or control mare was present in all bands during 2010–2017. Likewise, the composition of adult mares in each band, as well as the band stallions, remained relatively stable during this period. By the end of the 2017 foaling season, 14% (4/29) of treated mares and 11% (3/28) of control mares had died of various causes (e.g., malnutrition, broken appendage, dystocia, unknown causes). Except for these mares and one vaccinated mare that was not re-captured at the 2013 gather, all others were observed for foaling and other field measurements for all eight years of this investigation.

We met our sampling objective by observing more than 95% of all mares weekly (and sometimes more often) from 1 March to 1 August each year of the study. It is possible that some foals were born and died without being detected but given the intensity of the sampling observations, we feel that this was highly unlikely. Observations during the remainder of the year and following winter were less intense and more opportunistic depending upon available personnel, weather, and road conditions. During this time, mortality of foals was more likely to have gone undetected.

### Vaccine effectiveness

#### Primary vaccination (2009–2013)

**Fig 1:**
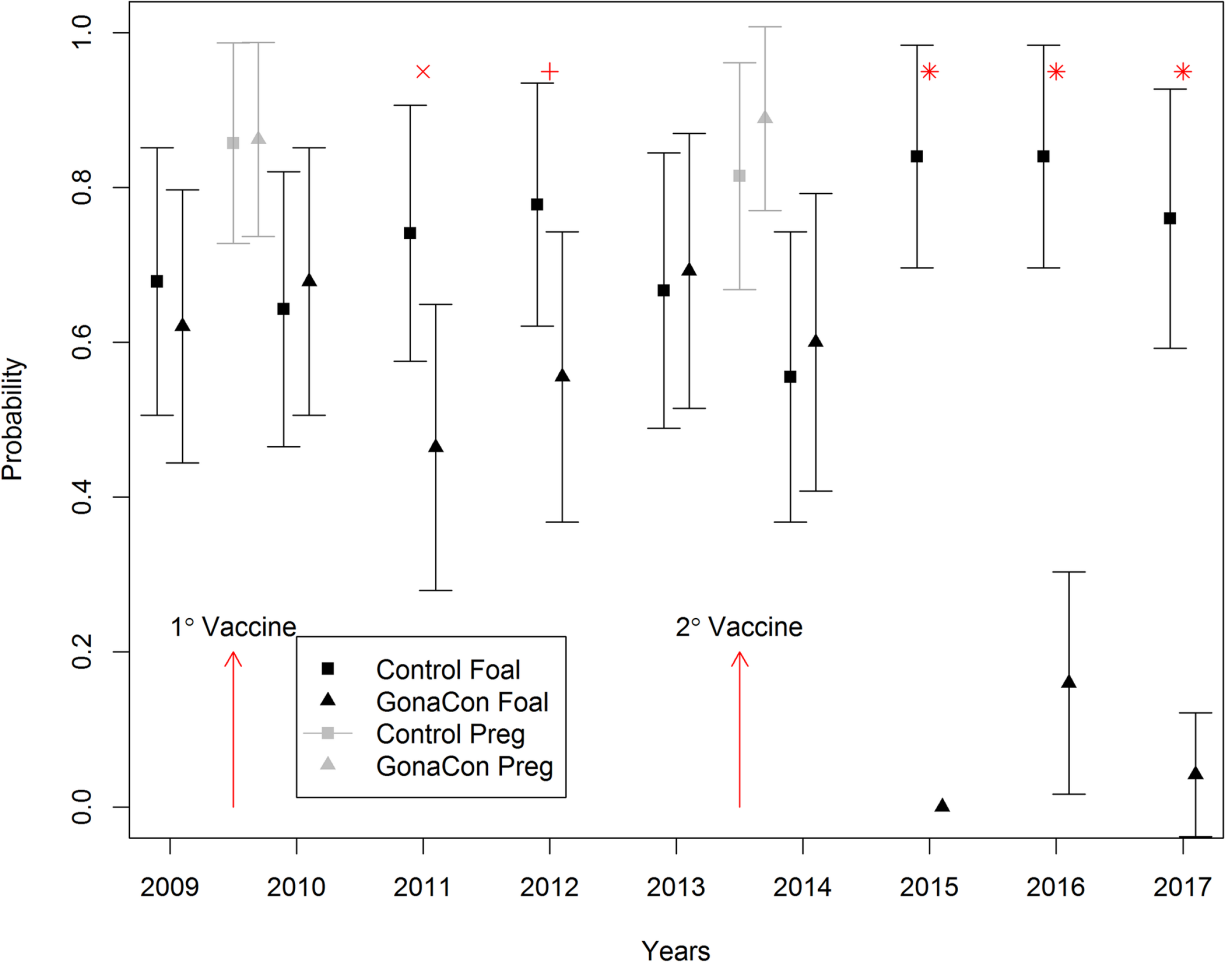
Comparative probability of foaling and pregnancy for treatment and control groups of free-ranging feral horses (*Equus caballus)* mares selected for this experiment. Mares were treated with a primary vaccination of GonaCon-Equine in October 2009 and then reimmunized with the same vaccine in September 2013 at scheduled gathers at Theodore Roosevelt National Park, North Dakota, USA. GonaCon vaccinations occurred at the time points represented by the red arrows. Symbols correspond to observed p-values for relative risk comparisons between treatment groups within years (p-value between 0.05 and 0.1 = +, for < 0.05 = x, and for < 1x10^-05^ = *).

Mean foaling proportions of treated (0.62 (18/29) 95% CI = 0.44–0.79) and control (0.68 (19/28) 95% CI = 0.50–0.85) mares during the 2009 pre-treatment foaling season were not different (*P =* 0.65) indicating that prior to contraception, treatment groups exhibited equal fertility [S1 Table]. Further evidence was provided by individual mares at the 2009 gather and primary vaccine inoculation. The proportion of treated (0.86 (25/29), 95% CI = 0.71–0.95 and control (0.85 (24/28), 95% CI = 0.70–0.95) mares determined to be pregnant, via transrectal ultrasonography, were not different (*P* = 0.63) [S1 Table, Fig 1]. This provided an opportunity to compare the effects of GonaCon-Equine vaccination on the existing pregnancy of treated mares and neonatal health and survival to that of untreated control mares. Foaling proportions of treated (0.68 (19/28) 95% CI = 0.50–0.85) and control (0.64 (18/28), 95% CI = 0.46–0.82) mares during 2010 were not different (*P =* 0.78) (Fig 1). Births occurred from early March to early September with 97% (35/36) observed during the first four months of the foaling season (1 March to 1 June). Average foaling dates in 2010 for treated and control mares were 5 May (95% CI = 22 April–18 May) and 10 May (95% CI = 25 April–25 May), respectively. No foal was detected for 12 mares (6 treated: 6 control) that were determined to be pregnant at the 2009 gather. None of these mares showed evidence of pregnancy during the intensive foaling period or for the remainder of the year. We surmised that most of these foals were either aborted or died as neonates between the periods from 20 October 2009 (gather) to 1 March 2010 (beginning of foaling observations). Regardless of timing or cause of death, the proportion of mares that foaled in 2010 underestimated the proportion of mares that were determined to be pregnant at the 2009 gather by 24% for treated mares and 21% for control mares.

Estimated age of all foals at first observation was 2.4 days (95% CI = 1.7–3.1days). Most neonates (97%), from both experimental groups, were classified as vigorous and in good to excellent condition when first observed. Neonatal survival rate from parturition to 14 days of age was estimated to be 0.95 (18/19, 95% CI = 0.75–0.99) for foals born to GonaCon-treated females and 0.88 (16/18, 95% CI = 0.64–0.98) for foals born to control mares (*P =* 0.54). After 14 days of age, post-neonatal survival rates (14–200 da) averaged 0.97 (30/31, 95% CI = 0.84–0.99) and were similar for both experimental groups (*P =* 0.57). These results support our prediction (*H*_*3*_*)* that inoculation with GonaCon-Equine vaccine, during approximately the second trimester of pregnancy, does not affect the existing pregnancy of treated females or neonatal health and survival.

The proportion of treated mares that foaled (13/28) following a single vaccination was lower than that for control mares (19/26) for the second (2011) (*P =* 0.04) and third (15/27 vs 21/27) (2012) (*P* = 0.08) post-treatment foaling seasons but was similar (18/26 vs (18/27) (*P* = 0.67) to control mares for the fourth (2013) season, demonstrating reversibility of the primary vaccine treatment (Fig 1). Even though we observed a significant reduction in foaling proportions between treated and control mares during 2011 and a declining effect in 2012, therapeutic effectiveness and relative risk reduction estimates were low to modest and estimated to be 0.37 (95% CI = 0.01–0.60) and 0.28 (95% CI = -0.06–0.51), respectively (Table 1). These findings lend support to our hypotheses *(H*_*1*_*)* that a single vaccination with GonaCon-Equine is reversible and suppresses fertility for multiple years post-treatment in a portion of treated animals but with diminished effectiveness over time.

**Table 1:** Comparative relative risk reduction (RRR), 95% confidence intervals, and p-values associated with differences in foaling proportions between GonaCon-treated and control mares during 2009–2017.

Year	Relative Risk Reduction	95% Confidence Interval		p-value
	(RRR)	Lower	Upper	
2009	0.0852	0.3757	-0.3402	0.6500
2010	-0.0555	0.2750	-0.5301	0.7797
**2011**	**0.3732**	**0.6028**	**0.0109**	0.0381*
2012	0.2857	0.5178	-0.0581	0.0861
2013	-0.0384	0.2826	-0.5032	0.8430
2014	-0.08	0.3216	-0.7194	0.7482
**2015**	**1**	**1**	**NA**	2.57E-09*
2016	0.8095	0.9236	0.5247	1.94E-06*
2017	0.9451	0.9920	0.6217	4.15E-07*

*Significant p-values (<0.05).

#### Secondary vaccination (2013–2017)

At the scheduled gather in October 2013, we extended our evaluation of GonaCon-Equine by assessing the effects of revaccination on fertility and safety in these same experimental mares treated four years after the primary vaccination. Evidence of similar fertility for individual mares was demonstrated at the 2013 gather, where pregnancy proportions of treated (0.92 (23/25), 95% CI = 0.75–0.98) and control (0.88 (23/26), 95% CI = 0.71–0.96) mares were similar (*P* = 0.86) [S1 Table]. Except for one treated and one control mare, all others had conceived and given birth to at least one foal during 2009–2013. For the 2013 foaling season, foaling proportions of treated (0.69 (18/26), 95% CI = 0.51–0.87) and control (0.66 (18/27) 95% CI = 0.49–0.84) mares were not different (*P* = 0.84) providing additional evidence that treatments groups were of equal fertility prior to reimmunization (Fig 1).

Like 2010, mean foaling proportions during the first post-treatment foaling season (2014) were not different (*P* = 0.74) between treated (0.60 (15/25), 95% CI = 0.41–0.79) and control (0.56 (15/27), 95% CI = 0.37–0.74) mares (Fig 1) supporting similar observations in 2010 that revaccination could be applied to pregnant mares, during mid-gestation, without risk to the existing pregnancy. Foaling date distribution was comparable to that observed in 2010 following the primary vaccination. Average foaling date for treated mares was estimated to be 27 April (95% CI = 5 April– 20 May) and 19 April (95% CI = 6 April– 2 May) for controls. No foal was observed for 15 mares (8 treated: 7 control) that were determined to be pregnant at the 2013 gather. Like 2010 estimates, foaling proportions underestimated pregnancy proportions determined at the 2013 gather for both treated and control mares by approximately 30% and 34%, respectively (Fig 1). These data, together with similar observations in 2010, support the inference that foaling proportions are not an accurate proxy for pregnancy proportions but provide a limited but practicable field measurement for determining contraceptive effectiveness. Average foal age at first observation across both treatment and control groups was 2.6 (95% CI = 1.5–3.3) days. Nearly all foals born to revaccinated and control mares were classified as vigorous and found to be in good to excellent condition when first observed.

Neonatal survival rate to 14 days of age for foals born to revaccinated mares was 0.87 (13/15), 95% CI = 0.62–0.96 and 0.93 (14/15), 95% CI = 0.70–0.98) for foals born to control mares (*P* = 0.49). After 14 days of age, post-neonatal survival rates were 0.80 (12/15), 95% CI = 0.55–0.92) for revaccinated mares and 0.73 (11/15), 95% CI = 0.48–0.89) for control mares (*P =* 0.55). These results reflect similar findings following a primary vaccination with GonaCon-Equine and reinforces the deduction (*H*_*3*_*)* that reimmunization is safe for treatment of pregnant females and does not affect neonatal or post-neonatal health or survival when applied at approximately mid-gestation.

Unlike results from the single vaccination trial, we observed, not only highly significant reduction in foaling proportions between treated and control mares following reimmunization but also a remarkably effective contraceptive response. Except for the first foaling season following treatment application, (2014) in which the vaccine was not expected to have an effect (*P* = 0.75), foaling proportions in reimmunized mares were lower (*P <*0.001) than that for control mares for all subsequent years (2015–2017) (Fig 1). This was particularly evident for the second post-treatment foaling season (2015) when none 0.00 (0/25), 95% CI = 0.0) of the reimmunized mares produced a foal while the proportion of control mares foaling was estimated to be 0.84 (21/25, 95% CI = 0.69–0.98). During the third post-treatment foaling season (2016), four treated mares produced a foal resulting in a foaling proportion of 0.16 (4/25), 95% CI = 0.01–0.30) while the proportion of control mares foaling was identical to that observed in 2015 (Fig 1). These foals were determined to be vigorous and in good to excellent condition at birth, however, two of these foals, born in September, were not observed the following spring and were categorized as post-natal mortalities and presumed to have died during winter (2016/2017).

In 2017, no additional treated mares produced a foal or showed evidence of pregnancy. However, one of the treated mares that had foaled in 2016 died of apparent natural causes (age-related malnutrition) during 2017 and two other revaccinated mares that had foaled in 2016 failed to produce a foal that year resulting in a foaling proportion of 0.041 (1/24), 95% CI = 0.03–0.12) (Fig 1). The foaling proportion for mares in the control group (2017) was 0.84 (21/25, 95% CI = 0.69–0.98) and higher (*P <*0.001) than that for GonaCon-treated mares (Fig 1). It should be noted that the apparent decrease in foaling proportions in GonaCon-treated mares from 2016–2017 and resulting increase in vaccine effectiveness (Table 1) is likely due to the inherent error associated with the small sample size (*n* = 4) of mares in this treatment group that regained fertility. Overall, there was both a substantial decrease in foaling proportions (Fig 1) and an exceedingly high level of effectiveness (Table 1) for treated mares compared to controls for 3 years post-revaccination (2015–2017) (P <0.001). Thus, fertility measurements during 2015–2017 support our prediction (*H*_*2*_) that revaccination with GonaCon-Equine would be more effective in suppressing foaling proportions in treated females compared to controls than a single immunization (Fig 1, Table 1).

### Side effects

#### Behavioral

We collected behavioral data on 73 feral horses (22 males, 25 treated females, 26 saline females) for 218.3 h in 2014. The median age of observed stallions was 12 years (range = 9–19 years), median age of observed control females was 8 years (range = 7–20), median age of observed treated females was 9 years (range = 7–22), and median band size was 8 horses (range = 2–14). There were no differences detected between treatment groups in any time budget behavior category (Table 2). As band size increased, feeding decreased 1.24% (95% CI = 0.48–2.00) per additional horse in the band. Likewise, locomotion increased 0.20% (95% CI = 0.07–0.33) and maintenance decreased 0.10% (95% CI = 0.01–0.19) per additional horse in the band.

**Table 2:** Treatment and supported effects in a mixed-effects linear regression of feral horse (*Equus caballus*) time budget behaviors (e.g. feeding, resting, locomotion, maintenance, social) and all-occurrence social behaviors (e.g. herding, reproduction, agonism, harem- social) at Theodore Roosevelt National Park, USA. Variance for the random effects of time of day (j) and individual horse identity (k) are shown as σ_j_^2^ and σ_k_^2^.

Behavior	Effect	*t*	*P*	Difference	95% confidence limit		σ_j_^2^	σ_k_^2^
					Lower	Upper		
**Feeding**	Treatment	-0.125	0.900				0.004	0.003
	Band Size	-3.193	0.001	-0.012	-0.020	-0.005		
**Resting**	Treatment	0.590	0.555				0.001	0.001
**Locomotion**	Treatment	-0.143	0.886				<0.001	<0.001
	Band Size	3.047	0.002	0.002	0.001	0.003		
	Foal Presence	2.900	0.004	0.012	0.004	0.021		
**Maintenance**	Treatment	-1.193	0.233				<0.001	<0.001
	Band Size	-2.238	0.025	-0.001	-0.002	-0.001		
**Social**	Treatment	-0.037	0.970				<0.001	0.001
** **	Foal Presence	2.499	0.013	0.027	0.006	0.049		
**Herding**	Treatment	-0.909	0.368				0.009	<0.001
**Reproduction**	Treatment	1.555	0.159				<0.001	<0.001
**Agonism**	Treatment	0.669	0.528				<0.001	0.048
**Harem-social**	Treatment	2.620	0.012	0.140	0.033	0.247	0.007	<0.001

Foal presence influenced locomotion, with barren females moving 1.24% (95% C I = 0.40–2.08) more than females with dependents. Foal presence also influenced the social behavior component of time budgets, with barren females interacting with others 2.74% (95% CI = 0.59–4.90) more than females with dependents.

Variance among individuals had little influence on any of the behaviors modeled (Table 2). Variance was also minimal between time periods of observation; however, there were some significant differences in amount of activity by time of day. An estimated 6.88% (95% C I = -0.73–14.5) more feeding occurred in the 1601–2000 h time-period than did earlier in the day, and this was reciprocated by an estimated 3.33% (95% CI = 1.09–7.79) less resting, 0.34% (95% CI = 0.15–0.82) less maintenance, and 1.30% (95% CI = 0.51–3.11) less social behavior during the same period.

There were no differences detected between treatment groups in herding, reproduction, or agonism, but treatment group did influence harem-social behavior. Observed instances of harem-tending behavior provided too few data to model. Because these social behaviors were not as dependent on other broad categories as is the case with compositional time budgets [51], we re-estimated the social behavior models with only treatment and supported effects to allow for clearer interpretation of the results.

Stallions initiated harem-social behavior 13.9% (95% CI = 3.25–24.68) less toward control females than toward treated females. Though all harem-social records were analyzed as a group, it should be noted that 55.8% of the 308 harem-social events were sub-categorized as allogrooming. While the significant difference between treatment groups was detected, the variance among individuals for this behavior was near zero (Table 2).

#### Physiological

No study mares exhibited antibody titers to any of the infectious diseases that were surveyed for (i.e., equine herpesvirus-1, equine infectious anemia, equine viral arteritis and contagious equine metritis) thus eliminating this factor as a potential cause of infertility in GonaCon-treated females.

No control mares, treated with saline, showed any evidence of injection site reactions. Swelling and discharge were never observed in this group. Likewise, these mares showed no evidence of lameness or gait abnormalities in either hind limb. Consistent with our hypothesis (*H*_*3*_*)*, approximately 72% of treated mares (21/29) displayed a visible reaction at the site of injection after a single vaccination with GonaCon-Equine (S1 Photo). A single mare developed a draining abscess after the initial vaccination. These lesions were persistent over multiple years. At the time of the 2013 roundup and revaccination, 81% (21/26) of vaccinated mares continued to have palpable swelling at the original site of vaccine injection.

Like initial vaccination reactions, during the first-year post-revaccination, approximately 50% (13/26) of mares continued to show swelling on the left hip at the site of the 2009 injection and 50% developed a reaction on the right hip at the site of revaccination in 2013. Two of these new reactions were draining abscesses. Yet again, injection site reactions were persistent with approximately half of the mares with swellings at one or both injection sites, 3 years after revaccination. None of the GonaCon-treated mares displayed any evidence of lameness, altered gait or abnormal range of movement throughout the 8 years they were observed.

While body condition varied between individuals and study years, it did not vary between treatment groups (*P* = 0.14) over the course of the study. Likewise, there was no effect of presence of a foal on body condition (*P* = 0.16). Average body condition ranged from 3.7–4.9 (moderately thin to moderate body condition) for all study animals over the 8 years that mares were observed. Individual body condition scores ranged from 1–7.

## Discussion

### Reproduction

This study demonstrated that a single vaccination against GnRH, using GonaCon-Equine, administered during mid-gestation, was safe, initiated short duration (2 yrs.) infertility in some mares, and was reversible, but was minimally effective in reducing fertility of treated females compared to controls. For two foaling seasons following vaccine treatment, we observed statistically significant reductions (28–38%) in foaling proportions of treated versus control mares but no effect by the third-year post-treatment, thus confirming the reversibility of the vaccine.

These results parallel similar findings from other experimental evaluations of GonaCon-Equine reported for captive and free-ranging mares. In a comparable study in Nevada with feral horses in a natural environment, GonaCon-Equine reduced foaling proportions by an average of 33% over a 3-year period but, like our study, contraception was only modestly effective over this period [23]. In contrast, contraceptive effectiveness of captive mares treated with GonaCon was greater and longer lasting (≥ 4yrs) than either of these studies [22]. The disparity between captive and free-ranging animals in contraceptive response to GonaCon vaccine is not limited to feral horses but has also been observed between captive and free-ranging white-tailed deer [14, 18, 20] and elk [15, 16, 19]. Although these investigations did not suggest a definitive causation for these differences, they all pointed to suppressed and less persistent GnRH antibody concentrations in free-ranging ungulates compared to their captive counterparts suggesting a relatively compromised or weakened immune response to the vaccine that resulted in reduced contraceptive effectiveness.

It is widely acknowledged that differences in vaccine effectiveness can be attributed to increased environmental stressors (i.e., nutritional status, injuries, parasite load, pathogen exposure, and social dynamics) that can inhibit a more vigorous immune response in free-ranging animals in a natural environment [59, 60]. It follows that while efficacy trials with captive animals can provide an important first approximation of vaccine safety and performance under controlled conditions; they may offer only limited inference to free-ranging animals that are not buffered against natural stressors that may decrease immune response and vaccine effectiveness. Regardless of the factor(s) contributing to the limited effectiveness of GonaCon in free-ranging animals, it appears that the immune response from a single vaccination does not consistently provide multiple years of infertility in all or even a high proportion of these animals.

In comparison to a single inoculation with GonaCon-Equine, the effect of reimmunization on foaling proportions was highly significant which allowed clear differentiation between treated and control mares for multiple breeding seasons. Compared to a single vaccination, reimmunization of mares in this study resulted in a much higher (58%) average vaccine effectiveness (range = 0.80–0.94) than the single vaccination for a 3-year period (2015–2017). Likewise, this level of effectiveness following reimmunization was on average higher than that previously reported for free-ranging mares treated with a single application with GonaCon-Equine [23] and 32% above what was reported for captive mares treated with the same vaccine formulation [22]. These results support the conclusion that a booster immunization with GonaCon-Equine can provide a highly effective, multi-year suppression of fertility in free-ranging horses and these results may be consistent in other animal species, as well.

It is fundamental knowledge that a secondary response to a vaccine generally results in a more rapid production of antibodies that are produced in greater amounts and over a longer time compared to the primary vaccination [25]. Repeat immunizations using a variety of GnRH vaccines in domestic horses have been shown to improve contraceptive efficacy. However, unlike commercially available short duration vaccines (< 1 yr.) developed for domestic horses [29, 61], GonaCon-Equine is formulated by combining a non-biodegradable oil in water-based emulsion and an optimum concentration of immunostimulatory killed mycobacteria to form a depot usually deep in muscle tissue. This depot injection is thought to allow for a slow release and prolonged stimulation so that the formulation can act for much longer periods of time (years) than is possible with standard injections (months). This effect is thought to be responsible for the extended antibody response of 3–4 years in vaccinated deer [14, 18, 20, 62], elk [15, 16], and horses [22].

While this response was not unexpected, the magnitude and duration of effectiveness of GonaCon-Equine following revaccination, even 4 years after the initial vaccination, is salient and relevant to the management of fertility in free-ranging horses. First, it demonstrates that a booster vaccination can stimulate a highly effective immune response that can result in multiple years (≥ 3 yrs.) of contraception. Second, it provides an initial reference point for defining the optimum revaccination schedule required for long-term reproductive management of female horses in a natural environment. And finally, it supports the consideration that while a single application may be preferred from a practical management perspective, GonaCon-Equine is more effective, in free-ranging horses, if repeat vaccinations are delivered on a periodic basis. While initial results are encouraging, additional research is needed to complete the objectives of this study including: 1) to define the duration of effective contraception post-revaccination, 2) to determine if long-term or permanent infertility is a possible outcome, and 3) to assess if return to fertility (if it occurs) results in altered birth phenology of treated mares. We will investigate these questions over the next three years of this study. Additionally, there may be a more optimal revaccination schedule which allows for altered duration of effectiveness or is more conducive to management schedules.

### Side effects

After revaccinated in October 2013, time budget and social behaviors of mares in spring/summer of 2014 were comparable to those observed during the same period in 2010, following the initial treatment in October 2009. We found no evidence of differences in frequency or intensity of social behaviors including estrous behavior associated with treatment. Both treatment and control groups displayed few estrus behaviors in either 2010 [37] or during 2014. Behaviors associated with estrus were observed only 17 times in treated and 57 times in control mares out of 1148 observed social behavior events. This supports our earlier findings that pregnant mares rarely show overt estrous-related behaviors and similarly GonaCon-Equine treated mares only occasionally display these behaviors, although each for different reasons. Once a mare is pregnant, progesterone likely subverts much of the estrous type behavior that would generally be displayed with high estrogen levels, and only occasionally do domestic horses display and stand for mounting when pregnant [63]. Relatively small amounts of estrogen are secreted as follicles develop and then regress. In the absence of progesterone, relatively small amounts of estrogen are likely sufficient to induce erratic estrous behavior as was observed in these mares. However, the small amounts of estrogen were likely insufficient to induce an LH surge and subsequent ovulation.

Regardless of the underlying endocrinology associated with these behaviors, vaccinated and control mares both displayed social interactions that maintained herd structure; herding, tending, and defending behaviors from the stallion; and social hierarchies. The only meaningful factor that influenced the amount of time spent in social behaviors (e.g. allo-grooming, herding and tending) was the presence or absence of a foal. Mares with foals spent more time alone with the foal than those without off-spring, which is to be expected given their social and nutrient requirements during the neonatal and post-natal periods [50]. It is possible that long-term absence of foals could influence social behavior on a longitudinal scale, but additional studies are needed to investigate such phenomena on an appropriate time scale.

Other techniques for reducing the fertility of free-ranging species, such as vaccination with the native porcine zona pellucida vaccine (PZP) and tubal ligation, maintain the competency of the endocrine aspects of fertility. This can lead to unintended consequences with repeated estrous cycling in polyestrous species. In fact, in a population of white-tailed deer, where most reproductive females had received tubal ligations, fawning was negligible; however, there was more than a 700% increase in the number mature males attracted to the area occupied by a high number of estrous cycling females [64]. Similarly, PZP vaccination has extended the length and intensity of breeding seasons in horses [49, 65–68], deer [69, 70], and elk [71]. GonaCon-Equine may avoid these inadvertent consequences by functionally inducing mimicry of pregnancy in females which continues to be an important part of the social structure of the group but does not invite intense adverse breeding behaviors.

Researchers have generally hypothesized that by alleviating the energetic demands of gestation and lactation, contracepted females will attain improved body condition over pregnant females that require additional food resources to produce and rear an offspring. However, for free-ranging large ungulates, empirical evidence supporting [72] or refuting [73–75] this prediction is limited and equivocal. In this investigation, contracepted mares that experienced no gestation and lactation did not exhibit improved body condition over mares that successfully reproduced. Individual mares in each experimental group, attained an average BCS of 5.0 (moderate) or better, which has been reported to be the minimally optimal level of stored fat necessary to achieve maximum reproductive efficiency during pregnancy and lactation [53, 76]. These levels of body condition were reflected in the high proportion of pregnant mares (0.85–0.92) observed in each treatment group at the management roundups in 2009 and 2013.

We acknowledge that our sampling intensity and/or sensitivity of our ocular index to body condition may not have enabled us to detect fine-scale differences between experimental groups. However, we conducted these evaluations during time periods when differences in body condition between pregnant and non-pregnant (GonaCon-treated) females should have been the greatest. Namely, during early spring (March) when fats deposits are depleted over winter and during April–August when the energetic demands of late gestation and lactation are increasing.

The body condition of an animal is dependent on a balance between energy intake and expenditure. When intake is not sufficient to meet energy requirements for various activities (i.e. maintenance, growth, activity, gestation, lactation, etc.), fat reserves and eventually lean body tissue will be lost. The fact that pregnant and lactating mares in this study were in similar body condition to that of contracepted ones suggest that food is unlikely a limiting factor for free-ranging horses at THRO. This is primarily due to the conservative management of multiple species of ungulates and their food resources [77–79]. The consequence of this approach is that only under the most extreme climatic conditions, such as prolonged drought, will forage be limiting to herbivores at THRO, regardless of reproductive status.

The only detectable adverse side effect of vaccination was intramuscular swelling at the vaccination site. Mares treated with GonaCon-Equine consistently showed evidence of inflammatory reactions at the injection site. While we never observed lameness associated with this reaction, several mares revealed draining abscesses within one-year post-vaccination. This is consistent with results for other wild ungulates treated with the same or similar GonaCon vaccines [13, 15, 34]. Given the designed highly inflammatory nature of both the adjuvant, which contains killed mycobacteria and non-biodegradable oil, as well as, the foreign protein carrier molecule, these types of reactions are predictable. In fact, they are likely necessary for optimum vaccine efficacy [80]. It is impossible to assess the total impact of these lesions on animal welfare; however, in this investigation, these did not have a measurable effect on body condition, locomotion, or social behaviors. Therefore, until additional research suggests otherwise, we conclude that the presence of injection site lesions following GonCon vaccination do not pose a serious contraindication associated with the application of this vaccine, and there appear to be minimal long-term effects on individual animal welfare.

## Conclusions

Controlling abundance of wildlife species that are simultaneously protected, abundant, competitive for resources, and in conflict with some stakeholders is a formidable challenge for resource managers. We demonstrated that the GnRH vaccine, GonaCon-Equine, could be an effective immunocontraceptive for free-ranging feral horses, particularly when the primary vaccination is followed by reimmunization four years later. This vaccine was shown to be safe for pregnant females and neonates and did not result in deleterious behavioral side effects during the foaling/breeding season. The only adverse reactions to vaccination were non-debilitating inflammatory responses at injection sites. One noteworthy implication has emerged regarding long-term management of free-ranging horse populations using GonaCon-Equine vaccine: effective management and development of population models will need to incorporate repeat immunizations of this vaccine to optimize management strategies aimed at stabilizing the growth rate of feral horse populations. Our research suggests that practical application of this vaccine in feral horses will require an initial inoculation that may provide only modest suppression of fertility followed by reimmunization over time that together could result in greater reduction in population growth rates. Future research will begin to define the most effective revaccination schedule with GonaCon-Equine for suppressing reproductive rates in free-ranging horses, the duration of effectiveness, and the return to fertility following treatment. Moreover, applying GonaCon-Equine to control the growth of feral horse populations will require that resource managers choose specific tactics for treating animals. Choices must be made on the number and age to treat and the frequency of treatment needed to maintain the desired population age structure and genetic diversity. Decisions on the most beneficial tactics will depend on overarching management goals and long-term objectives for the population.

## Supporting information

S1 Table Comparative metrics and pregnancy proportions, by age class, for treatment and control groups of free-ranging mares selected for this experiment. (PDF)

S1 Photo An Intramuscular swelling (raised area of tissue) observed in a Theodore Roosevelt National Park horse, 9 months after hand-injection with GonaCon-Equine vaccine. (TIF)

## References

[1] Woodroffe R, Thirgood S, Rabinowitz A. People and Wildlife: conflict or coexistence? Cambridge University Press; 2005.

[2] Diamond J. Evolution, consequences and future of plant and animal domestication. Nat 2002;418: 700–707. 10.1038/nature01019 12167878

[3] Asa C, Porton I. The need for wildlife contraception. In: Asa C, Porton I, editors. Wildlife contraception Issues, methods, issues, and applications; 2005 pp. xxv–xxxii.

[4] Caughley G. Overpopulation. In: Jewell P, Holt S, editors. Problems in management of locally abundant wild mammals New York: Academic Press; 1981 pp. 7–19.

[5] Hone J. Wildlife damage control Victoria: CSIRO; 2007.

[6] Olsen S. The roles of humans in horse distribution through time In: Ransom JI, Kaczensky P, editors. Wild equids: ecology, management, and conservation. Baltimore: Johns Hopkins University Press; 2016 pp. 105–120.

[7] Wagner F. 48th North American Wildlife and Natural Resources Conference. In: Status of wild horse and burro management on public rangelands. Logan: The College; 1983. p. 116–133.

[8] Garrott R, Oli M. A Critical Crossroad for BLM's Wild Horse Program. Science. 2013;341: 847–848. 10.1126/science.1240280 23970685

[9] National Research Council. 2013 Using science to improve the BLM wild horse and burro program: a way forward Washington, D. C: National Academy of Science; p. 1–398.

[10] Clarke I, Cummins J. The temporal relationship between gonadotropin releasing hormone (GnRH) and luteinizing hormone (LH) secretion in ovariectomized ewes. J. Endocrinol 1982;111: 1737–1739. 10.1210/endo-111-5-1737 6751801

[11] Hazum E, Conn P. Molecular mechanism of gonadotropin-releasing hormone (GnRH) action. I. The GnRH receptor. Endocr Rev 1988;9: 379–86. 10.1210/edrv-9-4-379 2851440

[12] Molenaar G, Lugard-Kok C, Meloen R, Oonk R, Koning J, Wessing C. Lesions in the hypothalamus after active immunization against GnRH in the pig. J Neuroimmunol 1993; 48: 1–11. 8227303 10.1016/0165-5728(93)90052-z

[13] Curtis P, Richmond M, Miller L, Quimby F. Physiological effects of gonadotropin-releasing hormone immunocontraception on white-tailed deer. Hum Wildl Confl. 2008; 2: 35–46.

[14] Gionfriddo J, Denicola A, Miller L, Fagerstone K. Efficacy of GnRH immunocontraception of wild white-tailed deer in New Jersey. Wildl Soc Bull 2011;35: 142–148.

[15] Powers J, Baker D, Davis T, Conner M, Lothridge A, Nett T. Effects of gonadotropin-releasing hormone immunization on reproductive function and behavior in captive female Rocky Mountain elk (*Cervus elaphus nelsoni*). Biol of Reprod 2011;85: 1152–1160. 21753192 10.1095/biolreprod.110.088237

[16] Powers J, Monello R, Wild M, Spraker T, Gionfriddo J, Nett T, Baker D. Effects of GonaCon immunocontraceptive vaccine in free-ranging female Rocky Mountain elk (*Cervus elaphus nelsoni)*. Wildl Soc Bull 2014;38: 650–656.

[17] Baker D, Powers J, Oehler M, Ransom J, Gionfriddo J, Nett T. Field evaluation of the immunocontraceptive GonaCon-B in free-ranging horses (*Equus caballus*) at Theodore Roosevelt National Park. J Zoo Wildl Med 44: S147.

[18] Miller L, Gionfriddo J, Fagerstone K, Rhyan J, Killian G. The single-shot GnRH immunocontraceptive vaccine (GonaCon™) in white-tailed deer: comparison of several GnRH preparations. Am J Reprod Immunol 2008;60: 214–223. 10.1111/j.1600-0897.2008.00616.x 18782282

[19] Killian G, Kreeger T, Rhyan J, Fagerstone K, Miller L. Observations on the use of GonaCon^TM^ in captive female elk (*Cervus Elaphus*). J Wildl Dis 2009;45: 184–188. 10.7589/0090-3558-45.1.184 19204347

[20] Gionfriddo J, Eisemann J, Sullivan K, Healey R, Miller L, Fagerstone K et al Field test of a single-injection gonadotrophin-releasing hormone immunocontraceptive vaccine in female white-tailed deer. Wildl Res 2009;36: 177–184.

[21] Miller L, Rhyan J, Drew M. Contraception of bison by GnRH vaccine: A possible means of decreasing transmission of brucellosis in bison. J Wildl Dis 2004;40: 725–730. 10.7589/0090-3558-40.4.725 15650090

[22] Killian G, Thain D, Diehl N, Rhyan J, Miller L. Four-year contraception rates of mares treated with single-injection porcine zona pellucida and GnRH vaccines and intrauterine devices. Wildl Res 2008;35: 531–539.

[23] Gray M, Thain D, Cameron E, Miller L. Multi-year fertility reduction in free-roaming feral horses with single-injection immunocontraceptive formulations. Wildl Res 2010;37: 475–481.

[24] Baker D, Powers J, McCann B, Oehler M, Bruemmer J, Galloway N et al. Gonadotropin releasing hormone vaccine (GonaCon-Equine) suppresses fertility in free-ranging horses (Equus caballus): limitations and side effects of treatment. 8th International Conference on Wildlife Fertility Control. Washington, DC; 2002. p. 111.

[25] Tizard I. An introduction to veterinary immunology 2nd ed Philadelphia, PA: Saunders; 1982.

[26] Welborn L, DeVries J, Ford R, Franklin R, Hurley K, McClure K et al 2011 AAHA Canine Vaccination Guidelines. J Am Anim Hosp Assoc 2011;47: 1–42. 10.5326/jaaha-ms-4000 21998907

[27] Imboden I, Janett F, Burger D, Crowe M, Hässig M, Thun R. Influence of immunization against GnRH on reproductive cyclicity and estrous behavior in the mare. Theriogenology. 2006;66: 1866–1875. 10.1016/j.theriogenology.2006.04.038 16780942

[28] Elhay M, Newbold A, Britton A, Turley P, Dowsett K, Walker J. Suppression of behavioural and physiological oestrus in the mare by vaccination against GnRH. Aust Vet J 2007;85: 39–45. 10.1111/j.1751-0813.2006.00092.x 17300452

[29] Botha A, Schulman M, Bertschinger H, Guthrie A, Annandale C, Hughes S. The use of a GnRH vaccine to suppress mare ovarian activity in a large group of mares under field conditions. Wildl Res 2008;35: 548–554.

[30] Kirkpatrick J, Turner A. Achieving population goals in a long-lived wildlife species (*Equus caballus*) with contraception. Wildl Res 2008;35: 513–519.

[31] Kirkpatrick J, Lyda R, Frank K. Contraceptive vaccines for wildlife: a review. Am J Reprod Immunol 2011;66: 40–50. 10.1111/j.1600-0897.2011.01003.x 21501279

[32] Rutberg A, Grams K, Turner J Jr, Hopkins H. Contraceptive efficacy of priming and boosting doses of controlled-release PZP in wild horses. Wildl Res 2017;44: 174–181.

[33] Massei G, Koon K, Benton S, Brown R, Gomm M, Orahood D. Immunocontraception for managing feral cattle in Hong Kong. PLoS ONE. 2015;10: e0121598 10.1371/journal.pone.0121598 25856283 PMC4391848

[34] Gionfriddo J, Denicola A, Miller L, Fagerstone K. Health effects of GnRH immunocontraception of wild white-tailed deer in New Jersey. Wildl Soc Bull 2011;35: 149–160.

[35] Powers J, Baker D, Ackerman M, Bruemmer J, Spraker T, Conner M et al Passive transfer of maternal GnRH antibodies does not affect reproductive development in elk (*Cervus elaphus nelsoni*) calves. Theriogenology. 2012;78: 830–841. 10.1016/j.theriogenology.2012.03.033 22541328

[36] Killian G, Miller L, Ryan J, Dees T, Doten H. Evaluation of GnRH contraceptive vaccine in captive feral swine in Florida. Wildl Damage Management Conference. 2003. p. 128–133.

[37] Ransom J, Powers J, Garbe H, Oehler M, Nett T, Baker D. Behavior of feral horses in response to culling and GnRH immunocontraception. Appl Anim Behav Sci 2014;157: 81–92.

[38] Laird W. The geology of the South Unit of Theodore Roosevelt National Memorial Park. Theodore Roosevelt Nature and History Association. 1950;17: 225–240.

[39] National Oceanic and Atmospheric Administration (NOAA). 2002. National Climate Data Center (NCDC), Federal Building, Ashville, North Carolina, USA.

[40] Hanson P, Hoffman G, Bjugstad A. The vegetation of Theodore Roosevelt National Park, North Dakota: a habitat type classification. 1984. USDA Forest Service General Technical Report. Report No. RM–113.

[41] Bucca S, Fogarty U, Collins A, Small V. Assessment of feto-placental well-being in the mare from mid-gestation to term: transrectal and transabdominal ultrasonographic features. Theriogenology. 2005;64: 542–557. 10.1016/j.theriogenology.2005.05.011 15993936

[42] Drost M, Thomas P. Disease of the reproductive system In: Smith B, editor. Large Animal Internal Medicine 2nd Edition. 2nd ed Mosby-Year Book; 1996.

[43] Orenstein W, Bernier R, Dondero T, Hinman A, Marks J, Bart K, Sirotkin B. Field evaluation of vaccine efficacy. Bull World Health Organ 1985;63: 1055–1068. 3879673 PMC2536484

[44] Weinberg G, Szilagyi P. Vaccine epidemiology: efficacy, effectiveness, and the translational research roadmap. J Infect Dis 2010;201: 1607–1610. 10.1086/652404 20402594

[45] Dorai-Ray S. CRAN—Package binom [Internet]. Cran.r-project.org. 2014 [cited 31 March 2018]. Available from: https://CRAN.R-project.org/package=binom R package version 1.1

[46] Nakazawa M. CRAN—Package fmsb [Internet]. Cran.r-project.org. 2017 [cited 31 March 2018]. Available from: https://CRAN.R-project.org/package=fmsb R package version 0.6.0

[47] R Core Team (2015). R: A language and environment for statistical computing. R Foundation for Statistical Computing, Vienna, Austria. URL https://www.R-project.org/.

[48] Waring G. Horse behavior Noyes and William Andrew; 2003.

[49] Ransom J, Roelle J, Cade B, Coates-Markle L, Kane A. Foaling rates in feral horses treated with the immunocontraceptive porcine zona pellucida. Wildl Soc Bull 2011;35: 343–352.

[50] Ransom J, Cade B, Hobbs N. Influences of immunocontraception on time budgets, social behavior, and body condition in feral horses. App Anim Behav Sci 2010;124: 51–60.

[51] Ransom J, Cade B. Quantifying equid behavior–a research ethogram for free-roaming horses. 2009. U. S. Geological Survey Techniques and Methods 2-A9, 23p.

[52] Roelle J, Ransom J. Injection-site reactions in wild horses (Equus caballus) receiving an immunocontraceptive vaccine. 2009. U.S. Geological Survey Scientific Investigation Report 2009–5038, 15p.

[53] Henneke D, Potter G, Kreider J, Yeates B. Relationship between condition score, physical measurements and body fat percentage in mares. Equine Vet J 1983;15: 371–372. 6641685 10.1111/j.2042-3306.1983.tb01826.x

[54] Harville D. Maximum likelihood approaches to variance component estimation and to related problems. J Am Stat Assoc 1977;72: 320–328.

[55] DasGupta A, Cai T, Brown L. Interval estimation for a binomial proportion. Statist Sci 2001;16: 101–117.

[56] Bates D, Mäechler M, Bolker B, Walker S. Fitting linear mixed-effects models using lme4. J Stat Softw 2015;67: 1–48.

[57] Brinsko S, Blanchard T, Varner D, Schumacher J, Love C, Hinrichs K, et. al Manual of equine reproduction 3rd Edition Maryland Heights, Mo: Mosby/Elsevier; 2011.

[58] Card C, Hillman R. Parturition 1993. In: McKinnon AO, Voss JL. Equine reproduction. Philadelphia: Lea and Febiger; 1993 pp. 567–573.

[59] Demas G. The energetics of immunity: a neuroendocrine link between energy balance and immune function. Horm Behav 2004;45: 173–180. 10.1016/j.yhbeh.2003.11.002 15047012

[60] Houston A, McNamara J, Barta Z, Klasing K. The effect of energy reserves and food availability on optimal immune defence. Proc R Soc Lon B: Biol Sci 2007;274: 2835–2842. 10.1098/rspb.2007.0934 PMC2373797 17848371

[61] Garza F, Thompson D, French D, Wiest J, George R, Ashley K et al Active immunization of intact mares against gonadotropin-releasing hormone: differential effects on secretion of luteinizing hormone and follicle-stimulating hormone. Biol Reprod 1986;35: 347–352. 3094596 10.1095/biolreprod35.2.347

[62] Miller L, Fagerstone K, Wagner D, Killian G. The single-shot GnRH immunocontraceptive vaccine (GonaCon™) in white-tailed deer: comparison of several GnRH preparations. Human Wildl Confl 2009;3: 103–115. 10.1111/j.1600-0897.2008.00616.x 18782282

[63] Asa C, Goldfoot A, Ginther O. Assessment of the sexual behavior of pregnant mares. Horm Behav 1983;17: 405–413. 6662519 10.1016/0018-506x(83)90049-1

[64] Boulanger J, Curtis P. Efficacy of surgical sterilization for managing overabundant suburban white-tailed deer. Wildl Soc Bull 2016;40: 727–735.

[65] Powell D. Preliminary evaluation of porcine zona pellucida (PZP) immunocontraception for behavioral effects in feral horses (*Equus caballus*). J Appl Anim Welf Sci 1999;2: 321–335. 10.1207/s15327604jaws0204_6 16363936

[66] Nuñez C, Adelman J, Mason C, Rubenstein D. Immunocontraception decreases group fidelity in a feral horse population during the non-breeding season. Appl Anim Behav Sci 2009;117: 74–83.

[67] Nuñez C, Adelman J, Rubenstein D. Immunocontraception in wild horses (*Equus caballus*) extends reproductive cycling beyond the normal breeding season. PLoS ONE 2010;5: e13635 10.1371/journal.pone.0013635 21049017 PMC2964306

[68] Nuñez C, Adelman J, Carr H, Alvarez C, Rubenstein D. Lingering effects of contraception management on feral mare *(Equus caballus*) fertility and social behavior. Conserv Physiol 2017;5: cox018; 10.1093/conphys/cox018 29977561 PMC6007543

[69] Curtis P, Pooler R, Richmond M, Miller L, Mattfeld G, Quimby F. Comparative effects of GnRH and porcine zona pellucida (PZP) immunocontraceptive vaccines for controlling reproduction in white-tailed deer (*Odocoileus virginianus*). J Reprod Suppl 2002;60: 131–141. 12220153

[70] McShea W, Monfort S, Hakim S, Kirkpatrick J, Liu I, Turner J et al The effect of immunocontraception on the behavior and reproduction of white-tailed deer. J Wildl Manage 1997;61: 560–569.

[71] Heilmann T, Garrott R, Cadwell L, Tiller B. Behavioral response of free-ranging elk treated with an immunocontraceptive vaccine. J Wildl Manage 1998;62: 243–250.

[72] Turner A, Kirkpatrick J. Effects of immunocontraception on population, longevity and body condition in wild mares (*Equus caballus*). J Reprod Suppl 2002;60: 187–195. 12220158

[73] Miller L, Crane K, Gaddis S, Killian G. Porcine zona pellucida immunocontraception: long-term health effects on white-tailed deer. J Wildl Manage 2001;65: 941–945.

[74] Walter W, Kilpatrick H, Gregonis M. Does immunocontraception improve condition of free-ranging female white-tailed deer? J Wildl Manage 2003;67: 762–766.

[75] Conner M, Baker D, Wild M, Powers J, Hussain M, Dunn R et al Fertility control in free-ranging elk using gonadotropin-releasing hormone agonist leuprolide: effects on reproduction, behavior, and body condition. J Wildl Manage 2007;71: 2346–2356.

[76] Henneke D, Potter G, Kreider J. Body condition during pregnancy and lactation and reproductive efficiency. Theriogenology 1984;21: 897–909.

[77] Marlow C, Gagnon L, Irby L, Raven M. Feral horse distribution, habitat use, and population dynamics in Theodore Roosevelt National Park. 1992. National Park Service, Final Report, Contract 1200–9–C818, p. 36.

[78] Westfall J Jr., Irby L, Norland J. A forage allocation model for four ungulate species in Theodore Roosevelt National Park 1993 Montana State University, Bozeman, Montana, p. 57.

[79] Irby L, Norland J, Westfall J Jr, Sullivan M. Evaluation of a forage allocation model for Theodore Roosevelt National Park. J Environ Manage 2002;64: 153–169. 11995238 10.1006/jema.2001.0514

[80] Miller L, Fagerstone K, Eckery D. Twenty years of immunocontraceptive research: lessons learned. J Zoo Wildl Med 2013;44: S84–S96. 10.1638/1042-7260-44.4S.S84 24437088

