# Supplementary material for: Reimmunization increases contraceptive effectiveness of gonadotropin-releasing hormone vaccine (GonaCon-Equine) in free-ranging horses (Equus caballus): Limitations and side effects
Source: PLoS One. 2018 Jul 31;13(7):e0201570. doi: 10.1371/journal.pone.0201570 (PMC6067756; doi:10.1371/journal.pone.0201570)
Supplement: S1 Table — (PDF) [file pone.0201570.s001.pdf]

S1 Table. Comparative metrics and pregnancy proportions, by age class, for treatment and control groups of free-ranging mares selected for this experiment.

2009

Criteria	GonaCon-Equine		Control	
**Total sample size (n)**	29		28	
**2 yr**	5		6	
**3-4 yr**	9		9	
**5-10 yr**	13		11	
**>10 yr**	2		2	
**Body condition score:**	Mean	95% CI	Mean	95% CI
**2 yr**	4.8	3.6-5.9	4.7	4.3-5.1
**3-4 yr**	5.1	4.6-5.6	5.0	4.3-5.7
**5-10 yr**	5.5	5.2-5.9	5.1	4.6-6.5
**>10 yr**	5.0	4.2-6.5	5.5	4.5-6.5
**Body weight (kg):**				
**2 yr**	350	300-399	357	226-487
**3-4 yr**	438	407-468	411	379-442
**5-10 yr**	428	381-475	462	434-489
**>10 yr**	455	369-540	471	458-483
**Pregnancy proportion:**				
**2 yr**	0.8 (4/5)	0.49-1.00	0.67 (4/6)	0.35-0.94
**3-4 yr**	0.89 (8/9)	0.58-1.00	0.89 (8/9)	0.59-1.00
**5-10 yr**	0.92 (12/13)	0.77-1.00	0.91 (10/11)	0.53-1.00
**>10 yr**	1.00 (2/2)	0.34-1.00	1.00 (2/2)	0.34-1.00

**Overall**	0.86 (25/29)	0.71-0.95	0.85 (24/28)	0.70-0.95

2013

**Criteria**	**GonaCon-Equine**		**Control**	
**Total sample size (n)**	25		26	
**6-8 yr**	13		14	
**9-12 yr**	7		6	
**>12 yr**	5		6	
**Body condition score:**	Mean	95% CI	Mean	95% CI
**6-8 yr**	5.3	4.7-5.5	5.1	4.7-5.5
**9-12 yr**	5.7	4.8-6.2	5.7	5.0-6.3
**>12 yr**	5.2	4.5-5.9	5.4	4.8-6.0
**Body weight (kg):**				
**6-8 yr**	455	422-487	427	403-452
**9-12 yr**	451	404-499	459	404-513
**>12 yr**	450	381-516	427	354-499
**Pregnancy proportion:**				
**6-8 yr**	0.92 (12/13)	0.67-0.92	0.86 (12/14)	0.60-1.00
**9-12 yr**	0.86 (6/7)	0.49-0.97	0.89 (8/9)	0.59-1.00
**>12 yr**	1.00 (5/5)	0.56-1.00	1.00 (6/6)	0.30-0.91
**Overall**	0.92(23/25)	0.75-0.98	0.88 (23/26)	0.71-0.96
