# Supplementary figures and images for: Reimmunization increases contraceptive effectiveness of gonadotropin-releasing hormone vaccine (GonaCon-Equine) in free-ranging horses (Equus caballus): Limitations and side effects
Source: PLoS One. 2018 Jul 31;13(7):e0201570. doi: 10.1371/journal.pone.0201570 (PMC6067756; doi:10.1371/journal.pone.0201570)

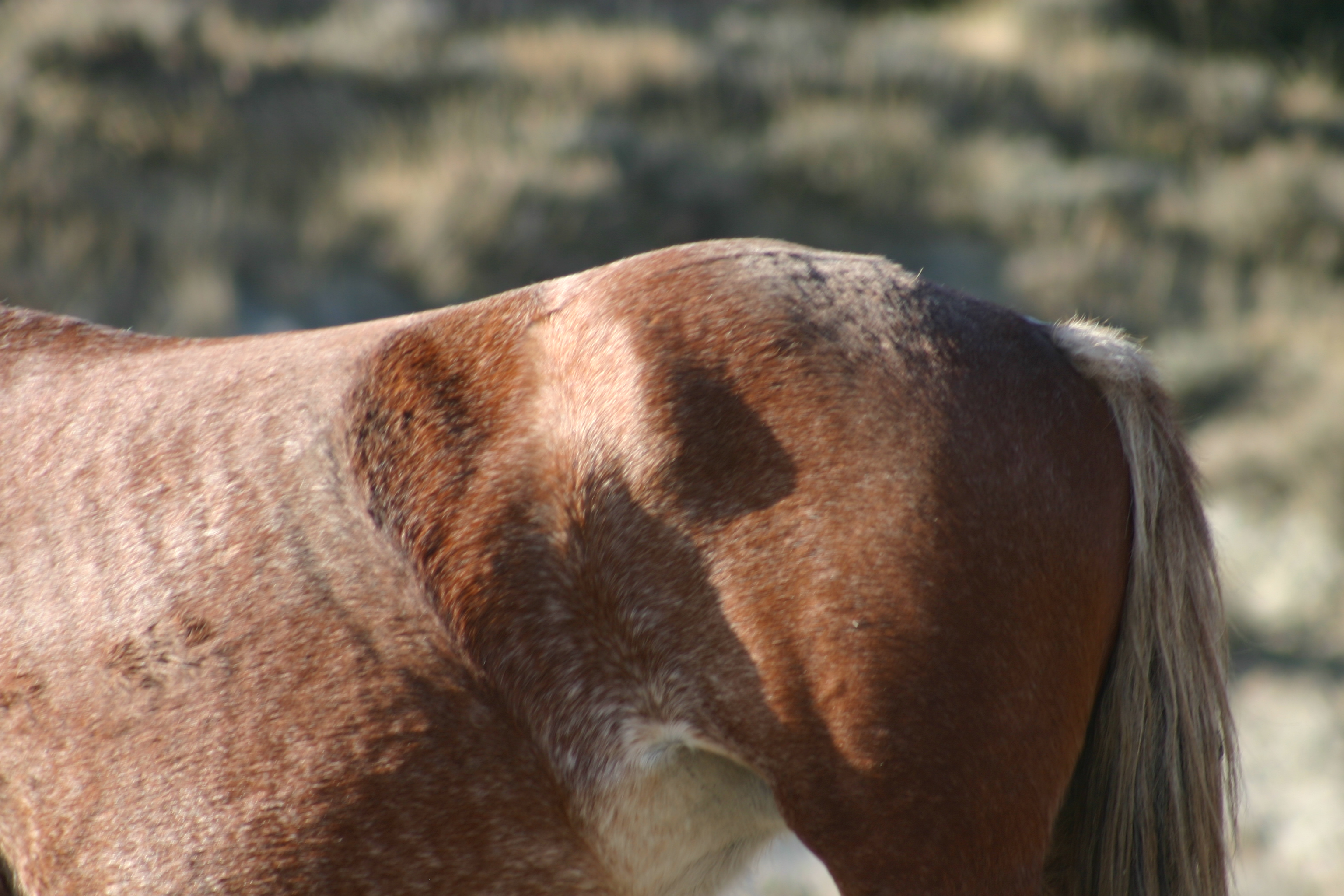

Supplement: S1 Photo — (TIF) [file pone.0201570.s002.tif]
